# Reliability of the Star Excursion Balance Test with End-Stage Knee Osteoarthritis Patients and Its Responsiveness Following Total Knee Arthroplasty

**DOI:** 10.3390/jcm13216479

**Published:** 2024-10-29

**Authors:** Bodor Bin Sheeha, Ahmad Bin Nasser, Anita Williams, Malcolm Granat, David Sands Johnson, Omar W. Althomali, Nouf H. Alkhamees, Zizi M. Ibrahim, Richard Jones

**Affiliations:** 1 Department of Rehabilitation Sciences, College of Health and Rehabilitation Sciences, Princess Nourah Bint Abdulrahman University, P.O. Box 84428, Riyadh 11671, Saudi Arabia; bhbinsheeha@pnu.edu.sa (B.B.S.); nhalkhamees@pnu.edu.sa (N.H.A.); zmibrahim@pnu.edu.sa (Z.M.I.); 2 Department of Orthopaedics, College of Medicine, King Saud University, P.O. Box 145111, Riyadh 11362, Saudi Arabia; ahmadbn@mac.com; 3 School of Health and Society, University of Salford, Salford M6 6PU, UK; a.e.williams1@salford.ac.uk (A.W.); m.h.granat@salford.ac.uk (M.G.); r.k.jones@salford.ac.uk (R.J.); 4 Department of Orthopaedics, Stockport NHS Foundation Trust, Stockport SK2 7JE, UK; david.johnson@stockport.nhs.uk; 5 Department of Physiotherapy, College of Applied Medical Sciences, University of Ha’il, Ha’il P.O. Box 2240, Saudi Arabia

**Keywords:** dynamic balance, Star Excursion Balance Test, osteoarthritis, total knee arthroplasty, reliability

## Abstract

**Background/Objectives:** The Star Excursion Balance Test (SEBT) is a simple and feasible tool for assessing dynamic balance in individuals with knee osteoarthritis (KOA). It has an advantage as it replicates dynamic balance better than other static balance tools. This study aims to determine how reliable SEBT is among people with end-stage KOA, as well as how responsive it is and how well it correlates with performance-based outcome measures after TKA. **Methods:** Patients on the waiting list for TKA performed SEBT in the anterior, posteromedial and posteriorlateral directions twice within 7 days. The measurements were repeated 6 and 12 months after TKA. The participants completed performance-based outcome measurements (PBOMs) and the Oxford Knee Score (OKS) before and after TKA to estimate correlation. **Results:** In all directions, the intraclass correlation coefficient range (ICC) was 0.998–0.993, and there were no significant differences between the test and re-test mean SEBT scores. The standard error of measurement (SEM) ranged from 0.37% to 0.68%, and the minimum detectable change (MDC) ranged from 1.02% to 1.89%. The post TKA SEBT results show significant improvement, with a large effect size. There were large-to-medium correlations between SEBT and PBOMs before and after TKA, while OKS correlated only before surgery. The magnitude of change in SEBT, PBOMs and OKS did not correlate. **Conclusions:** SEBT is an extremely reliable tool for assessing dynamic balance in all three directions of severe KOA patients. It is sensitive enough to detect balance changes at 6 and 12 months post TKA. SEBT cannot be used to reflect the change in functional outcome improvement after TKA.

## 1. Introduction

Osteoarthritis (OA)—considered the most common type of arthritis—may affect the body’s joints [1]. Morphological changes in the joint structure include the synovial membrane, subchondral bone and cartilage [2]. While OA may affect small or large joints, the knee joint is most commonly affected [3]. Projections indicate that knee OA (KOA) affects over 527 million individuals worldwide [4,5,6]. In a recent study conducted in Saudi Arabia, the prevalence of symptomatic knee OA was found to be 18.9% among the general population with a higher prevalence among females and older population [1]. Various factors, such as gender, obesity, sports participation, genetic predisposition, prior injuries, occupational demands, and anatomical abnormalities like varus or valgus alignment, can contribute to the development of osteoarthritis (OA) [6,7]. Numerous conservative treatment options, such as exercise therapy, knee braces, foot orthoses, and transcutaneous electrical nerve stimulation (TENS), show efficiency in improving muscle strength and balance and reducing pain in the early stage of KOA, in addition to pharmacological options [8,9,10].

According to the International Classification of Functioning, Disability and Health (ICF) framework, KOA is a leading cause of impairment, limitation in activity and restrictions in participation [11]. Furthermore, OA is considered one of the main causes of physical disability among the general population [12,13]. Several factors can contribute to physical disability among individuals with KOA, which can lead to a reduction in quality of life and increase the risk of morbidity, and include a lack of functional capability and pain. Another possible cause of disability in individuals with KOA is a balance defect [14,15,16].

Balance is a crucial component of the activities of daily living, and impairments in balance increase the risk of falling [14,15,16]. Previous research has shown a higher prevalence of falling among individuals with KOA compared with non-OA subjects [17]. It has also been shown that KOA affects balance, and more so in those with severe KOA than with mild [8,18,19]. Balance is defined as the ability to control the center of gravity over the support’s base. Interestingly, previous studies have revealed that instability—a major factor associated with a high risk of falling and disability—is prevalent in individuals with end-stage KOA [20,21].

Dynamic balance, which closely mimics real-life physical activities, is crucial for assessment [22]. Furthermore, deterioration in dynamic balance is faster compared with static balance among individuals with KOA [19,23]. The step test, which is a commonly used method to assess dynamic balance [19,24], has two drawbacks: (1) it measures balance in only one direction (anteriorly), and (2) it neglects the distance between the step and the standing base. To monitor patients, clinics and researchers can use the Star Excursion Balance Test (SEBT)—a quick and inexpensive method for assessing dynamic balance [22]. During the test, the participant tries to stand on one leg and use the other leg to reach as far as possible in eight directions (anterior, anteromedial, medial, posteromedial, posterior, posterolateral, lateral, and anterolateral) [25]. To reduce redundancy in directions, previous studies have recommended performing the test in only three directions (anterior, posterolateral and posteromedial) [26,27], especially for individuals with KOA, as abductor and quadricep weakness have been shown to affect the activation pattern and proprioception [28].

SEBT has shown good reliability among healthy individuals and patients [29], but only one study has investigated SEBT’s reliability in the KOA population [30]. That study used the Altman classification criteria to diagnose OA, but radiological imaging did not confirm it. Interestingly, no previous study has investigated the reliability, and responsiveness of SEBT in end-stage KOA individuals. This is important because these individuals suffer from more balance defects than patients with mild OA. Therefore, the current study investigated the test–retest reliability of SEBT among people with end-stage KOA, in addition to correlating it with other recommended outcome measures, such as performance-based measurement tests (PBOMs) or Oxford Knee Scores (OKS), as well as its responsiveness in patients who were 6 and 12 months following total knee replacement (TKA). The findings may guide the clinician’s decision about the suitability of SEBT in terms of its reliability for a large population with end-stage KOA, its responsiveness in detecting the dynamic balance changes over the 12 months following the TKA using clinically feasible and non-costly tools, and its correlation with other recommended functional tests.

## 2. Materials and Methods

The current study assessed both the reliability and responsiveness of SEBT in addition to SEBT correlation with the recommended performance-based outcome measurements (PBOMs) and the Oxford Knee Score (OKS). The ethical approval was obtained from the Ethics Committees of Salford University (HSR1617-39, Approval date: 13 February 2017) and King Khaled University Hospital (E-17-2395, Approval date: 5 April 2017). The study was registered by ClinicalTrail.gov (NCT02998125). In all sessions, one assessor collected all measurements.

### 2.1. Participants

This study included patients diagnosed with radiologically unilateral KOA with a score of 4 on the Kellgren–Lawrence grading scale, who were scheduled for TKA. A score of 4 indicates end-stage OA, marked by severe sclerosis, narrowing joint space (usually bone end-on-bone end contact), bone contour deformity, and large, significant osteophytes. Patients who could not participate were those with bilateral knee TKA or unilateral knee revision, limited function due to musculoskeletal conditions other than unilateral KOA, neurological disorders, diabetes, unstable chronic diseases, advanced osteoporosis, heart disease or peripheral vascular disease. We excluded any participants who presented postoperatively with complications such as fracture, uncontrolled infection or deep vein thrombosis.

### 2.2. Sample Size Estimation

The sample size was calculated based on a previous study’s equation [31]. The calculation revealed that 35 participants were sufficient to show expected reliability, with a value of ρ1 ≥ 0.88, and acceptable reliability, with a value of ρ0 ≥ 0.70. We added two measurement sessions per participant to the equation, maintaining a power level of 90% and an alpha level of 0.05. To reduce the effect of a possible dropout, as the patients were to be followed for one year, 5% was added to the final calculation to reveal the 38 required participants.

### 2.3. Outcomes

#### 2.3.1. Star Excursion Balance Test

To conduct SEBT, we fixed three tape measures to the floor. One each was oriented anteriorly, posteromedially and posterolaterally, forming a 135° angle from the anterior angle. The participants were asked to stand on the affected side in the center of a grid and reach the contralateral leg as far as possible in the three directions. The test was conducted barefoot, and the participants were asked to bend the stance leg as far as possible while not raising the heel to be counted as a valid trial [29,32]. To reduce the learning effect, we completed four training trials before conducting three real trials [29]. The average of the three trials was normalized based on leg length measured from the anterior superior iliac spine to the medial malleolus in the supine position [32].

#### 2.3.2. Oxford Knee Score

The OKS is a 12-item self-reported questionnaire designed to assess pain and function after TKA surgery. It is not only short, reliable, valid and sensitive enough to detect changes but it also has an excellent response rate based on previous research [33,34,35,36]. The questionnaire has been ranked as the best scale for measuring the outcome of knee arthroplasty [37]. When translated into Arabic, the questionnaire demonstrated excellent psychometric properties [38]. Each item in the questionnaire was scored from zero to four, with zero being the worst. The questionnaire has a maximum value of 48 and a minimum value of 0.

#### 2.3.3. Performance-Based Outcome Measures

The Osteoarthritis Research Society International (OARSI) recommends using a set of PBOMs for the comprehensive assessment of individuals with KOA or hip OA [39]. Previous research has demonstrated good-to-excellent psychometric properties of the timed up-and-go (TUG) test, 6 min walk test (6MWT), 30 s chair stand test (30 s CST) and a stair climb test (SCT) in patients with TKA [40,41,42,43,44,45,46,47,48]. Therefore, these outcome measures were selected because they assess physical functions classified as activities when using the World Health Organization’s International Classification of Functioning, Disability, and Health (ICF) model, which has good psychometric properties and allows for the functional exploration of muscle strength, endurance, and balance [49]. We randomized the test order and provided 10 min of rest between each test to mitigate the effects of fatigue [47].

We conducted a 30 s CST using a chair that was 45 cm high. The participants were asked to start the test by sitting and crossing their arms on their chest, followed by standing fully and sitting completely, which they would repeat as fast as possible for 30 s. Two trials were conducted, and the mean of the two trials was used [40,50]. SCT measures the time required for the patient to ascend and descend a flight of 12 steps as quickly and safely as possible. A stopwatch was used to measure time. The height of each step was 18 cm and the depth was 28 cm. After conducting a practical trial, we allowed the participants to use the handrail if necessary [43]. TUG measures the amount of time needed to stand from a chair (45 cm height with armrest), walk for three meters, turn around and then return to the chair. The participants were allowed to use the armrest if needed and instructed to walk as quickly and safely as possible. After conducting the trials, we calculated the average of two trials, beginning when the patient leaned forward and ending when their hips touched the chair [43,51].

### 2.4. Surgical Intervention and Rehabilitation

All patients underwent TKA using a midline incision with a medial parapatellar approach. One of five surgeons (whoever was available) at the hospital performed each surgery. No patients had postoperative complications, and three types of prostheses were used, as follows: the Persona knee-replacement system (Zimmer, Inc., Warsaw, IN, USA), the NexGen^®^ LPS Flex cemented knee-replacement system (Zimmer, Inc.) and the Attune^®^ knee-replacement system (DePuy Synthes, a Johnson & Johnson Company, New Brunswick, NJ, USA). The hospitalization period spanned 5–6 days, during which we provided standard physical therapy in accordance with the hospital protocol to minimize confounding factors. On day one after the surgery, physical therapy aimed to mobilize the patients, encouraging them to be fully weight-bearing or full, if possible. During their hospital stay, each patient received exercises that focused on bed exercise, range of motion, lower limb strength and stair and gait training—a protocol that aims to achieve the patient’s maximum level of functioning. All patients underwent outpatient physiotherapy lasting at least one month (an average of three sessions per week), concentrating on lower-limb exercises, strengthening and gait/balance training.

### 2.5. Procedures

The study procedure was thoroughly elucidated to each prospective participant, and a consent form was secured following sufficient time for inquiries and deliberations. A preadmission session (T1) appointment was scheduled following each participant’s agreement. This visit was arranged to gather data for the balance evaluation of the initial session for test–retest reliability [52]. Seven days following the initial session (T2), a subsequent visit occurred, during which all outcome measures (SEBT, 30 s CT, 6MWT, SCT, TUG, and KOS) were gathered. The subjects then underwent total knee arthroplasty (TKA) and were scheduled for third (T3) and fourth (T4) visits at 6 and 12 months post-surgery, respectively. SEB, PBOM, and OKS were gathered during T3 and T4 to perform the correlation assessment [30].

### 2.6. Statistical Analysis

Statistical Package for Social Sciences (SPSS) software (Version 29) was used to conduct the analysis. The value of each outcome was entered into SPSS. To investigate the test–retest reliability intraclass correlation coefficient (ICC), a two-way mixed model was conducted between T1 and T2 with absolute agreement, and the selection of the model was based on the presence of one observer. The ICC yielded a numerical value between zero and one, which we interpreted using the following guidelines: 0.40–0.70, 0.70–0.90 and >0.90, indicating fair, good and excellent performance, respectively [53]. The standard error of measurement (SEM = standard deviation pooled $\times \sqrt{\left(1-\;\mathrm{ICC}\right)\;}$) [54] and minimum detectable change (MDC = (1.96 × $\sqrt{2}$ × SEM)) [55] were also calculated to indicate the level of disagreement between the measurements (absolute reliability) [56]. In contrast to the ICC, the SEM had a number with the same unit as the outcome measure. To further investigate the reliability, the mean difference between T1 and T2 was calculated using a paired sample *t*-test and a Bland–Altman plot [57].

The average of T1 and T2 for balance was calculated and then used to determine correlation and responsiveness. The correlation of the SEBT was determined by calculating the Spearman’s correlation coefficient (R) between the SEBT (absolute and change in normalized score) and the absolute and the change in value for OKS and all PBOM (SCT, 30 s CST, 6MWT and TUG). The interpretation of the correlation was based on the following criteria: small (R = 0.1–0.3), medium (R < 0.3–0.5) and large (R > 0.5). The responsiveness was assessed by detecting the ability of SEBT and other PBOMs (30 s CT, 6MWT, SCT, and TUG) and KOS to detect the difference between before the TKA and 6 and 12 months after the TKA (T3 and T4, respectively). A one-way repeated measures ANOVA, followed by a pairwise comparison, was used. Partial Eta squared was computed and used to represent the effect size (0.01 small, 0.06, medium and 0.14 large) [57].

## 3. Results

In total, 94 participants scheduled for TKA were invited to participate in the study, of which 56 did not meet the study criteria and 16 declined to participate. This led to 38 participants completing the first session (T1). The study lost three participants in T2, resulting in a final sample size of 35. The sample comprised 11 males and 24 females, with a mean age of 62 (±10.26) years and a mean leg length of 75.29 cm (±5.86).

**Figure 1:**
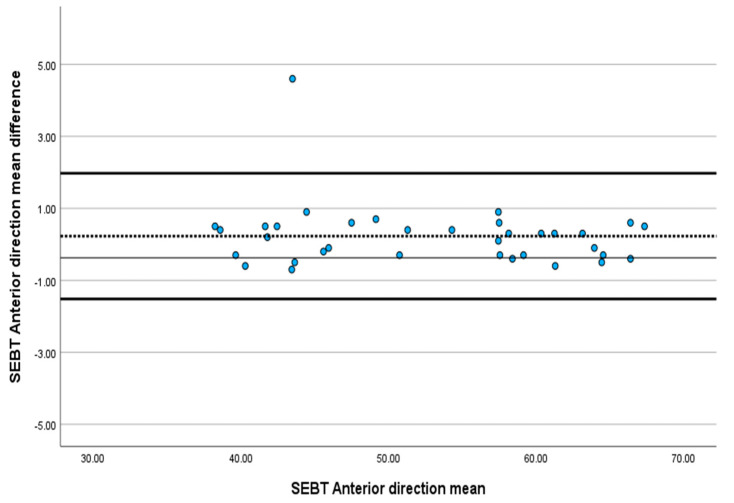
Bland-Altman plot showing the reliability of anterior direction SEBT.

**Figure 2:**
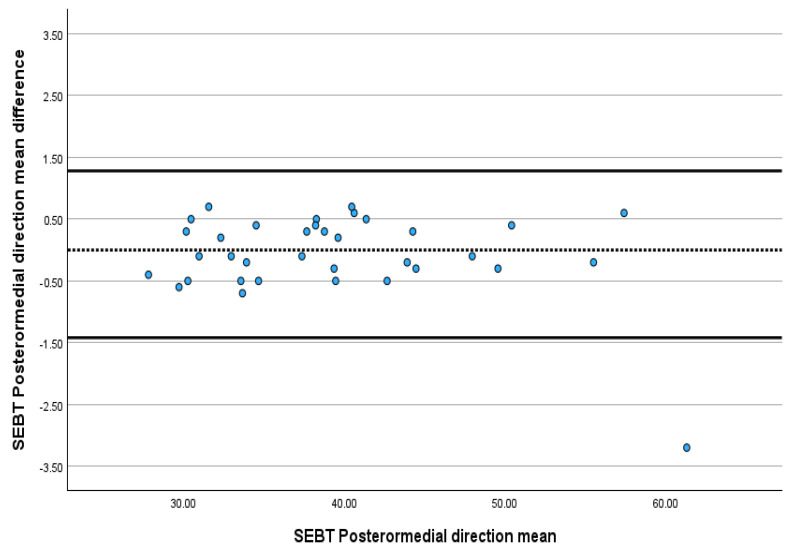
Bland–Altman plot showing the reliability of posterolateral direction SEBT.

**Figure 3:**
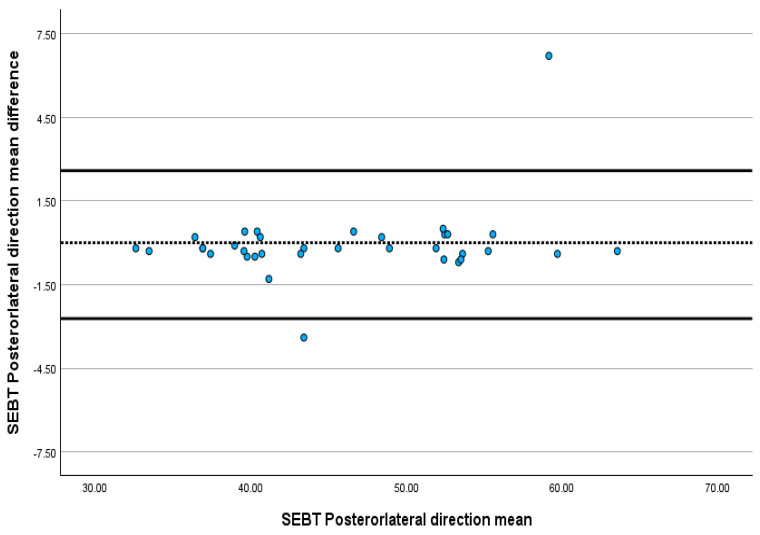
Bland–Altman plot showing the reliability of posteromedial direction SEBT.

The ICC value for SEBT in the anterior, posteromedial and posterolateral directions ranged between 0.993 and 0.998. Between T1 and T2, there was no significant difference between the SEBT values in the anterior, posteromedial and posterolateral areas, with a *p*-value greater than 0.05. The SEM for SEBT ranged from 0.37% to 0.68%, and the MDC ranged from 1.02% to 1.89% (Table 1). The Bland–Altman plots revealed that almost all SEBT scores in all directions were within the limits of agreement, indicating excellent absolute agreement (Figure 1, Figure 2 and Figure 3).

The correlation between SEBT in all directions and SCT, 30 s CST, 6MWT, TUG and KOS at T1 was significant (*p* < 0.5). The strength of the relationship between SEBT and SCT, 30 s CST, 6MWT, TUG and OKS varied from medium to large. In all directions, the correlation between SEBT and SCT, 30 s CST, 6MWT, TUG and KOS at T4 was significant in most of the outcomes (*p* < 0.5), except for KOS. The correlation strength was similar to T1, ranging from medium to large. There was no correlation between the magnitude of change in SEBT in all directions and the magnitude of change in SCT, 30 s CST, 6MWT, TUG and KOS (*p* > 0.5). (Table 2).

A repeated measures ANOVA showed a significant (*p* < 0.05) difference in SEBT in the anterior, posteromedial and posterolateral directions between timepoints. The test also revealed significant (*p* < 0.05) improvement from PBOM and KOS between the timepoints (Table 3). The results show a large effect size for all outcome measures. The pairwise comparison revealed significant improvement after surgery and with time between the measurement timepoints (Table 4).

## 4. Discussion

The current study assessed both the reliability and responsiveness of SEBT. The study also examined the correlation between SEBT and the recommended performance-based outcome measurements (PBOMs) and the Oxford Knee Score (OKS). The results generally indicate excellent reliability, responsiveness, and correlation with PBMs and OKS. These results contribute to the existing understanding that SEBT serves as a valuable instrument for assessing balance in both research and clinical settings among individuals with end-stage OA.

The results reveal excellent reliability for SEBT, ranging from ICC 0.998 to 0.993, which agrees with previous studies that have determined its reliability on healthy athletes and patients with anterior cruciate ligament [22,52,58,59,60]. This excellent relative reliability reflects SEBT’s ability to provide consistent results over time and differentiate between the performances of individuals scheduled for TKA. The *t*-test result, which revealed no significant difference (*p* > 0.05) between the test (T1) and the re-test (T2), supports the previous claim. The Bland–Altman plot, which helps measure the possible bias between the mean difference and the agreement intervals between the test and the re-test, showed that 97% of the SEBT scores in the anterior, posteromedial and posterolateral directions were within the limit of agreement. Despite the widespread use of SEBT for healthy individuals and those with different conditions, such as ankle sprains and anterior circulate ligament injuries, this is the first study to investigate its reliability among the end-stage KOA population.

As an outcome measure, SEM helps researchers and clinicians understand the amount of disagreement and, therefore, quantify real improvements. If the improvement passes the SEM then we can be assured, with 68% confidence, that it is a true improvement [61,62]. The results reveal that the SEM for SEBT ranged from 0.37% to 0.68%. Previous studies have shown similar values, ranging from 2.23% to 5.5% among healthy individuals [52,58,59] and from 2.68% to 3.08% among individuals with early-stage KOA [30]. MDC helps ensure that the researcher can be 95% confident that the change is real and has a clinical impact. The MDC values in the current study ranged from 1.02% to 1.89%, which is lower than previous studies with healthy participants [52,59]. The variation in healthy individuals with end-stage OA may explain this. End-stage OA patients can use SEBT due to its excellent absolute reliability and low values in MDC and SEM [63]. SEM and MDC can help researchers estimate the true expected change after treatment. For example, the clinician will be 68% (SEM) and 95% (MDC) certain that the change in SEBT anterior direction is real if it passes beyond 0.42% and 1.18%, respectively.

The SEBT results show significant improvement in all directions after 6 and 12 months in comparison with the pre-TKA results, and with a large effect size. The present study is the first to investigate SEBT responsiveness among individuals after TKA. Previous studies have investigated the sensitivity of SEBT among individuals diagnosed with KOA and showed significant improvement after 6 and 12 weeks of an exercise program, with a 0.74 effect size [30,64]. Several factors, including the nature of the treatment and the grade of the KOA, contributed to the smaller effect size, despite the BMI and age range being similar to the current study. The previous studies included individuals with grades 2 and 3 OA based on the Kellgren and Lawrence system [30,64], while the current study included end-stage OA. Individuals with anterior cruciate ligament deficiency showed a similar effect size (3.51), despite the inability to make a direct comparison [65]. In individuals with TKA, joint stability is an important factor that may affect balance, especially with muscle co-contraction and neuromuscular control differences in comparison with anterior cruciate ligament injury.

Regarding correlation in the current study, SEBT showed a medium-to-large correlation at all timepoints, which may indicate differences in constructive validity and may reflect that dynamic balance is a critical component of a task that requires locomotion. This indicates the importance of measuring balance among individuals with end-stage KOA and individuals with TKA. Except for T1, KOS (actual value and magnitude of change) did not show a significant correlation with SEBT. Furthermore, there was no correlation between changes in PBOM and SEBT magnitudes. KOS, being a patient-reported outcome, may not accurately reflect the magnitude of balance and function, potentially explaining the lack of agreement with SEBT [66]. This variation between outcomes and the poor correlation highlights the importance of each outcome in capturing different perspectives, either subjectively or objectively, if we aim to capture a holistic functional view.

It is important to consider the limitations of this study. The current study used only one observer to collect the test–re-test data, which reflects intra-rater reliability. Intra-rater reliability is important for providing valuable data, especially for follow-up when one observer is collecting the data. However, understanding inter-rater reliability is crucial, as it allows researchers and clinicians to understand the differences between them and make valid comparisons. Therefore, we recommend that future studies investigate inter-rater reliability. In the current study, the mean age was 62 (±9) years, involved unilateral knee involvements, and the research was carried out in one center. Therefore, future studies are recommended to investigate reliability in the older population, in bilateral knee involvements and should be carried out in multiple centers. This should be in addition to long-term investigations of sensitivity that occur 2–5 years following TKA, as this will allow for an exploration of the suitability of SEBT to detect the dynamic changes in the long term and in bilateral knee involvements following TKA.

## 5. Conclusions

In summary, people with KOA have a deficit in terms of their dynamic and static balance, and SEBT is a good outcome measure. However, before using any outcome measure in the clinic or in research, reliability and responsiveness must be determined. The current study showed that SEBT is reliable and sensitive enough to detect changes among individuals with end-stage OA. Furthermore, SEBT requires only one simple instrument (tape), which is placed on the floor, making it easy to use. The current study showed that SEBT can be used with people with advanced KOA. It has a low measurement error and can detect changes after TKA with a low minimal detectable change value. This emphasizes the value of such an outcome and supports its use.

## Figures and Tables

**Table 1:** Between-days reliability (ICC, SEM, MDC) for SEBT.

Outcomes (Percentage of Leg Length)	ICC (95% CI)	T1 Mean (SD)	T2 Mean (SD)	Mean Difference Between T1 and T2	*t*-Test Between T1 and T2	SEM	MDC
SEBT anterior direction	0.998 (0.995–999)	52.9 (9.4)	52.66 (9.56)	0.229	0.138	0.42	1.18
SEBT posteromedial direction	0.998 (0.997–999)	39.2 (8.1)	39.31 (8.1)	–0.069	0.560	0.37	1.02
SEBT posterolateral direction	0.993 (0.987–997)	46 (8.3)	46.02 (7.96)	–0.069	0.766	0.68	1.89

**Table 2:** Correlation between SEBT in all directions and SCT, 30 s CST, 6MWT, TUG and KOS.

Pearson Correlation			SCT	30 s CST	6MWT	TUG	OKS
Correlation baseline	SEBT anterior direction	Correlation coefficient	−0.653	0.52	0.469	−0.599	0.457
		Sig (2-tailed)	**<0.01**	**<0.01**	**<0.01**	**<0.01**	**<0.01**
	SEBT posteromedial direction	Correlation coefficient	−0.424	0.396	0.362	−0.449	0.505
		Sig (2-tailed)	**0.011**	**0.019**	**0.032**	**<0.01**	**<0.01**
	SEBT posterolateral direction	Correlation coefficient	−0.472	0.436	0.424	−0.488	0.570
		Sig (2-tailed)	**<0.01**	**<0.01**	**0.01**	**<0.01**	**<0.01**
Correlation post–TKA at test (T4)	SEBT anterior direction	Correlation coefficient	−0.670	0.404	0.0539	−0.432	0.074
		Sig (2-tailed)	**<0.01**	** 0.016 **	**<0.01**	**<0.01**	0.673
	SEBT posteromedial direction	Correlation coefficient	−0.435	0.453	0.421	−0.388	−0.055
		Sig (2-tailed)	**<0.01**	**<0.01**	** 0.012 **	** 0.021 **	0.754
	SEBT posterolateral direction	Correlation coefficient	−0.581	0.524	0.523	−0.409	0.038
		Sig (2-tailed)	**<0.01**	**<0.01**	**<0.01**	** 0.015 **	0.830
Correlation between the changes (T4–T1)	SEBT anterior direction	Correlation coefficient	−0.069	−0.019	−0.064	−0.165	−0.076
		Sig (2-tailed)	0.69	0.91	0.715	0.343	0.665
	SEBT posteromedial direction	Correlation coefficient	0.055	−0.103	−0.096	−0.058	−0.043
		Sig (2-tailed)	0.754	0.557	0.584	0.743	0.808
	SEBT posterolateral direction	Correlation coefficient	−0.011	0.181	−0.052	−0.10	0.001
		Sig (2-tailed)	0.948	0.229	0.769	0.566	0.993

**Table 3:** One-way ANOVA for the outcomes between timepoints.

Variable	Baseline	6 Months Post TKA	12 Months Post TKA	ANOVA	Partial Eta Squared
SEBT anterior direction	52.77 (9.47)	59.70 (9.45)	61.92 (9.33)	*p* < 0.01	0.695
SEBT posteromedial direction	39.28 (8.23)	44.03 (8.24)	45.72 (8.10)	*p* < 0.01	0.591
SEBT posterolateral direction	45.99 (8.11)	51.57 (8.23)	53.87 (7.91)	*p* < 0.01	0.777
SCT	54.46 (23.88)	39.11 (14.96)	32.00 (12.49)	*p* < 0.01	0.619
30 s CST	8.57 (2.65)	12.34 (2.21)	14.23 (1.70)	*p* < 0.01	0.850
6MWT	262.43 (83.43)	271.71 (83.58)	297.17 (80.23)	*p* < 0.01	0.587
TUG	17.66 (9.99)	12.63 (5.21)	9.11 (3.26)	*p* < 0.01	0.569
OKS	16.00 (6.24)	33.23 (3.65)	39.74 (1.56)	*p* < 0.01	0.918

**Table 4:** Pairwise comparison of the outcomes between timepoints.

Variable	Assessment Time	Comparison	Significant	Mean Difference
SEBT anterior direction	Baseline	6 months	*p* < 0.01	−6.926
		12 months	*p* < 0.01	−9.149
	6 months	12 months	*p* < 0.01	−2.223
SEBT posteromedial direction	Baseline	6 months	*p* < 0.01	−4.751
		12 months	*p* < 0.01	−6.443
	6 months	12 months	*p* < 0.01	−1.691
SEBT posterolateral direction	Baseline	6 months	*p* < 0.01	−5.580
		12 months	*p* < 0.01	−7.883
	6 months	12 months	*p* < 0.01	5.580
SCT	Baseline	6 months	*p* < 0.01	15.343
		12 months	*p* < 0.01	22.457
	6 months	12 months	*p* < 0.01	7.114
30s CST	Baseline	6 months	*p* < 0.01	−3.771
		12 months	*p* < 0.01	−5.657
	6 months	12 months	*p* < 0.01	−1.886
6MWT	Baseline	6 months	*p* < 0.01	−9.286
		12 months	*p* < 0.01	−34.743
	6 months	12 months	*p* < 0.01	−25.457
TUG	Baseline	6 months	*p* < 0.01	5.029
		12 months	*p* < 0.01	8.543
	6 months	12 months	*p* < 0.01	3.514
OKS	Baseline	6 months	*p* < 0.01	−17.229
		12 months	*p* < 0.01	−23.743
	6 months	12 months	*p* < 0.01	−6.514

## Data Availability

The raw data supporting the conclusions of this article will be made available by the authors on request.
